# Prevalence of *Trypanosoma cruzi* infection in dogs in the United States: a systematic review and meta-analysis of observational studies (2000–2024)

**DOI:** 10.3389/fvets.2025.1658198

**Published:** 2025-10-15

**Authors:** Victor Agbajelola, Walter Roachell, Joshua Bast, Paul Lenhart, Mauricio Solis, Ram K. Raghavan

**Affiliations:** 1 Department of Pathobiology and Integrative Biomedical Sciences, University of Missouri, Columbia, MO, United States; 2 Public Health Command West, Joint Base San Antonio (JBSA), San Antonio, TX, United States; 3 Entomological Sciences Division, Defense Centers for Public Health - Aberdeen, Aberdeen, MD, United States; 4 Public Health Command Central, Joint Base San Antonio (JBSA), San Antonio, TX, United States; 5 Department of Public Health, College of Health Sciences, University of Missouri, Columbia, MO, United States; 6 MU Institute of Data Science and Informatics, University of Missouri, Columbia, MO, United States

**Keywords:** American trypanosomiasis, canine Chagas infection, kissing bugs, *Trypanosoma cruzi*, United States, working dogs

## Abstract

**Background:**

Canine infection with *Trypanosoma cruzi*, the causative agent of Chagas disease, is a public health concern in the United States (U.S.), particularly in southern states where triatomine vectors are established. Dogs are considered important in the transmission cycle as potential reservoir hosts, with implications for both animal and human health. This study systematically reviewed observational studies to assess the prevalence, geographic distribution, and associated risk factors of canine *T. cruzi* infection in the U.S., and generated pooled prevalence estimates through meta-analysis.

**Methods:**

A systematic review and meta-analysis were conducted in accordance with PRISMA guidelines. Observational studies published between January 1, 2000, and March 31, 2024, were identified through searches of PubMed, Scopus, ScienceDirect, Web of Science, and Google Scholar, with the final search completed on April 30, 2024. Two reviewers independently extracted data, resolving discrepancies through consensus or consultation with a third reviewer. Prevalence estimates were pooled using a random-effects model, heterogeneity was quantified, and subgroup analyses and meta-regression were performed to explore sources of variability.

**Results:**

Sixteen studies comprised of 4,974 dogs across five states were included, and the pooled prevalence of canine Chagas infection was 12% (95% CI: 0.07–0.21), with significant heterogeneity (*I^2^* = 96%). Louisiana had the highest pooled infection prevalence (18%; 95% CI: 0.04–0.51). Highest pooled infection prevalence was found among working dogs (32%; 95% CI: 0.07–0.74), while shelter dogs had the lowest (7%; 95% CI: 0.04–0.12). The meta-regression indicated that the study year was significantly associated with canine *T. cruzi* infection prevalence (*p* < 0.001), with an estimated 11% increase in odds per year, suggesting either a real temporal rise or improved detection/reporting over time.

**Conclusion:**

This review confirms the presence of canine *T. cruzi* infection in the U.S., though evidence is limited to a few southern states and marked by methodological variability. Standardized diagnostics, clearer dog type classification, and concurrent vector surveillance are needed to improve reliability and expand the geographic scope of future estimates.

## Introduction

*Trypanosoma cruzi* infection is an important emerging concern in dogs in the United States (U.S.). Dogs are susceptible hosts for the parasite and can contribute to domestic transmission cycles, potentially serving as sentinels for human risk (1–3). Transmission occurs primarily through contact with infected triatomine insects (“kissing bugs”), either by vector feeding or ingestion, and the ecology of these vectors supports parasite circulation in both sylvatic and peridomestic settings (1). While the global burden of *T. cruzi* infection is concentrated in Latin America, where millions of people are affected (2–4), reports of infection in U.S. dogs have grown steadily over the past decades. The true burden of infection in dogs, however, remains poorly defined. This has important implications not only for pet health but also for high-value working populations, including military and law enforcement dogs, where infection and loss can lead to significant operational and economic consequences (5, 6).

The first reported cases of *T. cruzi* infection in dogs in the U.S. were documented in Texas between 1972 and 1975 (7). Since then, additional cases have been identified in multiple states, indicating a wider geographic spread than previously recognized (1, 8–12). Of particular concern is the vulnerability of both residential and working dogs, which frequently operate in outdoor environments and face elevated risks of exposure to infected vectors. Compromised health in these dogs not only affects their performance and health but also carries substantial zoonotic, economic and operational implications for both human health, national defense, law enforcement, and homeland security (5, 9, 10).

Clinical manifestations of *T. cruzi* infection in dogs range from asymptomatic cases to acute or chronic cardiac infections often leading to progressive myocardial inflammation, conduction abnormalities, and fibrin deposition in cardiac tissues, culminating in heart failure or sudden death in severe cases (3). These outcomes not only pose diagnostic and therapeutic challenges in veterinary medicine but also impact public health, because infected dogs may act as sentinels or potential reservoirs for human exposure, especially in environments where infected vectors (triatomine bugs) are present (1, 8).

Vector-borne transmission (either through contamination of a bite wound with infected triatomine feces or through oral ingestion of infected triatomines) remains the primary route of *T. cruzi* infection in dogs, with ecological conditions in the southern U.S., such as warm climate, abundant wildlife, and suitable habitats, favoring the survival and reproduction of competent triatomine vectors (3). These factors support sylvatic transmission cycles that may spill over into domestic settings, as suggested by the frequent detection of triatomine bugs near homes, kennels, and shelters, raising concerns about overlooked domestic transmission and increased exposure risk for dogs (5, 13).

There is growing concern that *T. cruzi* infection in dogs may extend beyond historically recognized areas of transmission (9, 14). Nonetheless, most documented cases remain concentrated in the southern U.S., particularly in states bordering Mexico, where established triatomine populations, cross-border ecological connections, and interactions with reservoir hosts sustain transmission cycles (15–18). Reports of canine infections in regions with low or undocumented triatomine activity (12, 19) further suggest that the distribution of infection may be underrecognized and that gaps remain in our understanding of *T. cruzi* epidemiology among different dog types across the country.

Given the zoonotic nature of *T. cruzi* infection, its economic implications, and national security concerns, there remain important knowledge gaps regarding its distribution and risk factors among different canine types, including residential, shelter, stray/feral, and working dogs. To address these gaps, this study systematically reviews observational research on canine *T. cruzi* infection in the U.S. from 2000 to 2024 and applies a meta-analytic framework to generate pooled prevalence estimates. The goal is not to make direct statistical comparisons between states or dog types with unequal sampling, but rather to synthesize fragmented evidence, highlight epidemiological patterns, and identify priorities for enhanced surveillance and future research. The findings aim to inform public health strategies, enhance disease surveillance, and guide interventions to mitigate the spread of *T. cruzi* among dogs and, by extension, humans. Specifically, our study aims to (1) estimate the overall prevalence of *T. cruzi* infection in dogs using pooled prevalence data from available studies, (2) assess geographic variations in prevalence across different U.S. states, and (3) assess pooled prevalence rates among different dog types (e.g., residential, shelter, working, and stray/feral dogs).

## Materials and methods

### Search and selection criteria

We included observational studies published between January 1, 2000, and March 31, 2024, that reported the prevalence of *T. cruzi* infection in dogs with data stratified at the state level. Eligible studies were required to use defined sampling methods and laboratory-confirmed diagnostic techniques. We then conducted a systematic review and meta-analysis of observational studies on Chagas disease in dogs in the U.S. between 2000 and 2024, following the Preferred Reporting Items for Systematic Reviews and Meta-Analyses (PRISMA) guidelines (20). A comprehensive literature search was performed using five electronic databases: PubMed, Scopus, ScienceDirect, Web of Science, and Google Scholar. The search strategy employed the following Boolean operators and keywords: (*“Trypanosoma cruzi”* OR *“Canine Chagas disease”*) AND (*“Dogs”* OR *“Canine”*) AND (*“Prevalence”* OR *“Detection”* OR *“Seroprevalence”*) AND (*“United States”* OR *“US”* OR *“USA”*). The search was limited to articles published in English, with the final search completed on April 30, 2024.

### Inclusion and exclusion criteria

Studies were eligible if they met the following criteria: (1) observational cross-sectional design conducted in the United States between January 1, 2000 and March 31, 2024; (2) reporting the prevalence of *T. cruzi* infection in dogs; (3) providing sufficient data to calculate prevalence, including both sample size and number of positive cases; and (4) employing serological and/or molecular diagnostic methods, with serological prevalence data stratified at the state level to enable geographic subgroup analyses. In cases where no serological estimates are available, we report the molecular prevalence, and where multiple serological prevalence estimates were reported using different diagnostic criteria, we extracted the stricter definition of positivity (i.e., confirmation by two assays) to ensure methodological consistency.

Studies were excluded if they were reviews, case reports, or experimental investigations; if they lacked sufficient prevalence data (e.g., missing sample size or number of positives); if they reported only aggregated national prevalence without state-level stratification; or if they relied solely on highly localized convenience samples unlikely to represent broader dog populations (e.g., single-clinic client-owned dogs). Also, studies that are not accessible online before April 2024 and those conducted outside the U.S. were excluded.

### Data extraction and quality assessment

From each included study, data were extracted on publication year, sample size, number of positive cases, diagnostic methods (serological), dog type (e.g., shelter, residential, working, stray/feral), and location (state) where the study was done. The initial search strategy and preliminary organization of information were informed by consultations with two graduate students. However, all data extraction, inclusion/exclusion decisions, classification, and final analyses were performed by the listed co-authors. Any discrepancies were resolved through discussion among the co-authors, with final validation of the dataset specifically performed by RR and VA to ensure accuracy and consistency. The methodological quality of the included studies was assessed using the Joanna Briggs Institute (JBI) Critical Appraisal Checklist for prevalence studies (21). Each study was scored on a 10-point scale, and those scoring seven or higher were considered to have a low risk of bias and were retained for meta-analysis.

### Selection of risk factors

The primary risk factors evaluated in subgroup and meta-regression analyses were dog type and state of study. These variables were selected because they were consistently reported across multiple studies and are supported in the literature as important determinants of *T. cruzi* transmission risk in dogs (1, 12, 15, 22, 23). Other potential variables, such as age, breed, sex, and management practices, were excluded from quantitative analyses due to inconsistent reporting across the included studies.

### Statistical analysis

Meta-analysis was performed using the *metafor* package in R (24). Effect sizes were calculated as the natural logarithm of the odds of infection, and variances were computed accordingly. A random-effects model using restricted maximum likelihood (REML) estimation was applied to account for between-study heterogeneity. Heterogeneity was assessed using Cochran’s Q test, the *I^2^* statistic, and tau-squared (τ^2^). Subgroup analyses were conducted to examine differences in prevalence by dog type and states. Meta-regression analysis was performed to assess the influence of potential moderators on prevalence estimates. Publication bias was evaluated using funnel plots and Egger’s regression test. Statistical significance was set at *p* < 0.05 for all analyses.

## Results

### Literature search and study selection

**Figure 1:**
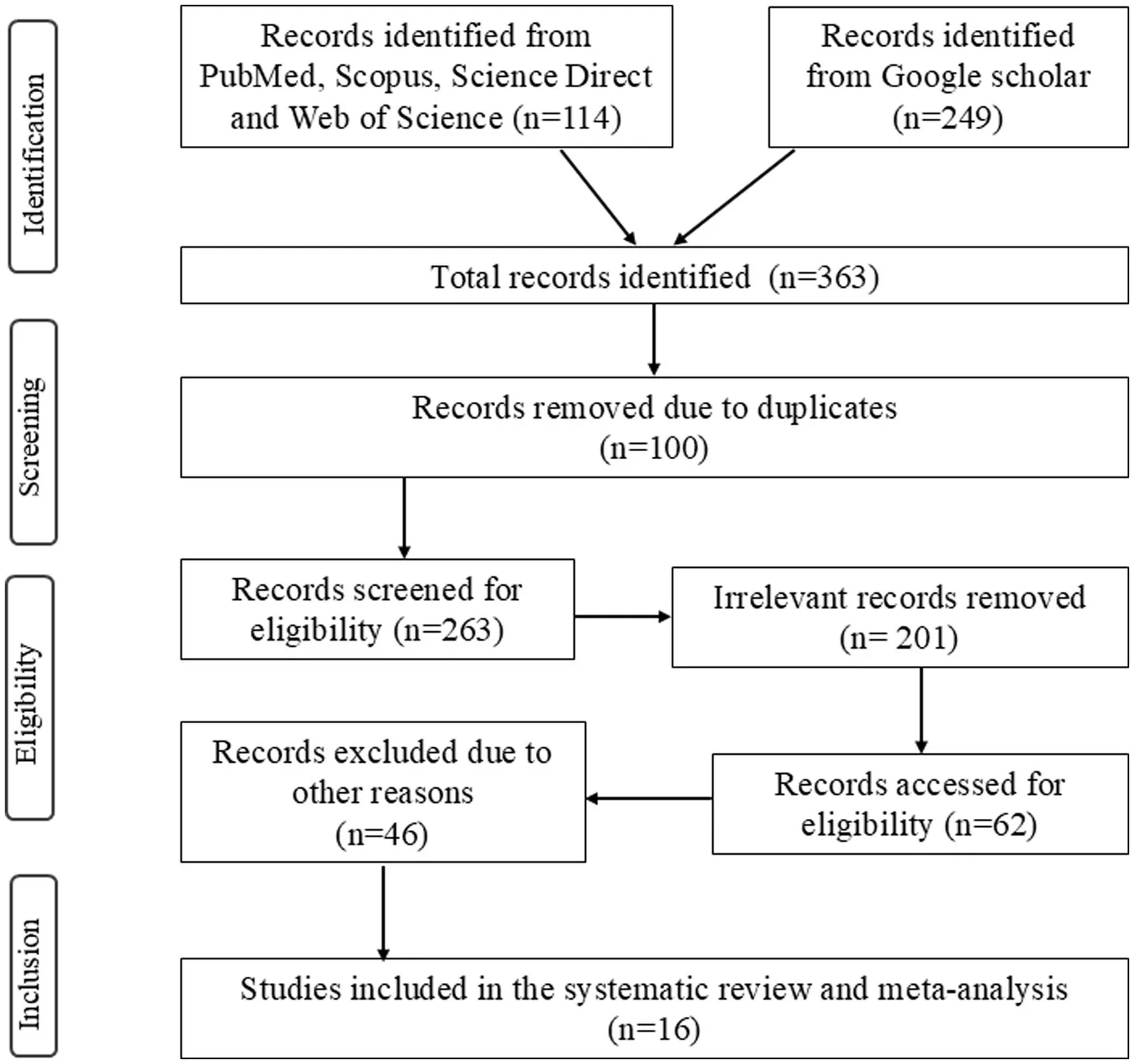
PRISMA flowchart of the article selection process.

The systematic literature search across five databases (PubMed, Scopus, ScienceDirect, Web of Science, and Google Scholar) yielded 363 publications. After removing duplicates (*n* = 100), the remaining articles (*n* = 263) were screened based on titles and abstracts. Of these, 201 articles were excluded due to lack of relevance (e.g., clinical trials, randomized studies, or non-epidemiological studies) or duplicated data. A total of 62 full-text articles were assessed for eligibility, with 46 excluded due to insufficient prevalence data or lack of clarity with regards to the number of positively diagnosed dogs. Ultimately, 16 studies met the inclusion criteria and were assessed/evaluated in the systematic review and meta-analysis (Figure 1).

### Study characteristics

A total of 16 studies (Table 1) met the inclusion criteria; however, 17 entries were ultimately included in the pooled meta-analysis due to methodological adjustments. In the study by Nieto et al. (11), dogs were originally grouped under a single “kennel” category. Based on reported housing and activity patterns, Group 1 dogs (hunting kennel) were reclassified as “working” dogs, while Group 2 dogs (residential pets) were reclassified as “residential” dogs. This allowed the study to contribute to two dog-type categories rather than being treated as a distinct category. Similarly, the study by Rodriguez *et al.* (25), which only reported molecular prevalence, was included by substituting its molecular prevalence estimate into the pooled analysis, as it was the sole study using molecular methods as the primary diagnostic outcome, providing state-level data from an otherwise underrepresented dog type (stray/feral).

**Table 1:** Characteristics of eligible observational studies included in the analysis.

Study/Year	Positive cases/sample size	Serological Prev (%)	Dog type	State	Bug Species
Allen and Lineberry, 2022 (37)	26/197	13.2	Shelter	Oklahoma	-
Curtis-Robles et al., 2017 (9)	49/85	57.6	Working	Texas	*T. gerstaeckeri, T. sanguisuga*
Curtis-Robles et al., 2018 (31)	4/14	28.6	Residential	Texas	*T. gerstaeckeri, T. rubida, T. protracta*
Elmayan et al., 2019 (35)	540/37	6.9	Shelter	Louisiana	-
Garcia et al., 2016 (18)	8/209	3.8	Shelter	Texas	-
Hodo et al., 2019 (17)	110/608	18.1	Shelter	Texas	-
Meyers et al., 2017 (10)	39/528	7.4	Working	Texas	*T. gerstaeckeri, T. rubida*
Nieto et al., 2009 Group 1^*^ (11)	16/31	51.6	Working	Louisiana	*T. sanguisuga*
Nieto et al., 2009 Group 2^*^ (11)	11/91	12.1	Residential	Louisiana	
Rodriguez et al., 2021^**^ (25)	49/95	-	Stray/feral	Texas	*T. gerstaeckeri, T. rubida, T. protracta*
Rosypal et al., 2010 (38)	1/90	1	Shelter	Virginia	-
Rowland et al., 2010 (39)	55/860	6.4	Residential	Tennessee	-
Shadomy et al., 2004 (40)	9/356	2.6	Residential	Texas	-
Tenney et al., 2014 (41)	18/205	8.6	Shelter	Texas	-
Curtis-Robles et al., 2017 (15)	66/209	31.6	Residential	Texas	*T. gerstaeckeri*
Beard et al., 2003 (8)	28/375	7.5	Stray/feral	Texas	*T. gerstaeckeri*
Bradley et al., 2000 (42)	11/301	3.6	Shelter	Oklahoma	*T. sanguisuga*

^*^ Nieto et al. (11): Group 1 dogs (hunting kennel) were reclassified as “working” dogs, and Group 2 dogs (residential pets) were reclassified as “residential” dogs.

^**^Rodriguez et al. (25): The only included study that used molecular methods as the primary diagnostic approach.

All studies included in the quantitative synthesis were cross-sectional investigations of *T. cruzi* infection in dogs conducted in the U.S. between 2000 and 2024, collectively sampling 4,974 dogs across five states (*n* = 5). Serological diagnostic techniques predominated, including enzyme-linked immunosorbent assay (ELISA), indirect fluorescent antibody test (IFAT), radioimmunoprecipitation assay (RIPA), and rapid immunochromatographic tests, while molecular methods (PCR) were employed less frequently. Nine (9) of the 16 studies reported triatomine species in or near study locations, most commonly *Triatoma sanguisuga*, *T. gerstaeckeri*, *T. rubida*, and *T. protracta*.

For analysis, dog types were categorized into four groups: (1) residential, (2) shelter, (3) working (including military, law enforcement, and hunting or service dogs), and (4) stray/feral (free-roaming, peridomestic, and unowned dogs captured for sampling). Initial classifications of “feral” and “stray” were merged into a single category to reflect overlapping definitions across studies. In Rodriguez et al. (25), “feral” dogs were specifically defined as free-ranging, unowned animals captured through field trapping, distinct from “shelter” dogs admitted to facilities such as animal control or humane societies. We acknowledge that the composition of shelter populations is heterogeneous and may include former feral dogs, owner surrenders, and strays later reclaimed by owners.

### Prevalence of canine *Trypanosoma cruzi* infection in the U.S.

**Figure 2:**
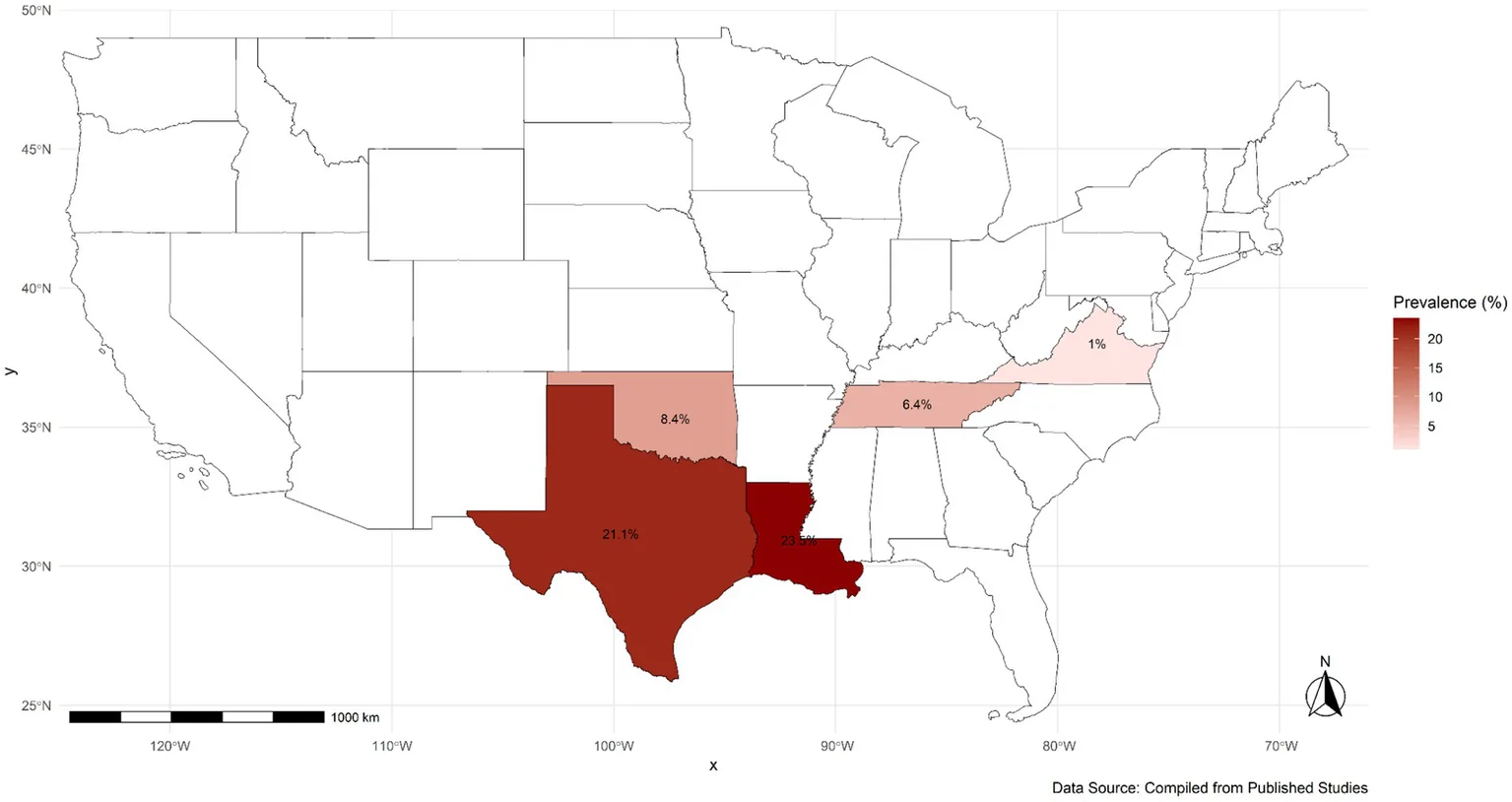
Geographic distribution of the mean infection prevalence of *T. cruzi* infection in dogs in the U.S.

Across the 16 included studies, a total of 4,974 dogs were tested, of which 537 were positive for *T. cruzi*, yielding an overall raw prevalence of 10.8%. Considerable variation in infection prevalence was observed across states and dog types, reflecting ecological and epidemiological heterogeneity in transmission risk (Table 2). The highest mean infection prevalence estimates were reported from Louisiana (23.5%; 95% CI: 0.04–0.51) and Texas (21.1%; 95% CI: 0.07–0.30). While these findings suggest a concentration of reported canine infections in parts of the southern U.S. (Figure 2), they should be interpreted cautiously, as data were not uniformly available across all states within the region. Vector data were also reported in several studies, with a total of 785 triatomine bugs identified across included investigations, distributed in Texas, Oklahoma, and Louisiana. No vectors were reported in the published studies conducted in Tennessee and Virginia. This observation aligns with regional differences in entomological surveillance but was not analyzed further in the pooled estimates.

**Table 2:** Meta-analysis of the Prevalence of *T. cruzi* infection in dogs in the U.S. between 2000 and 2024.

Study variables	Positive cases/sample size	Mean seroprev. (%)	Pooled prev. (%)	95% CI	Cochran’s Q	*I* ^2^	df	*p* value
Total infection	537/4974	18.0	12.5	0.07–0.21	435	96.3	15	<0.01
States								
Texas	380/2684	21.1	15	0.07–0.30	290.8	97	9	<0.01
Oklahoma	37/498	8.4	7	0.02–0.23	13.9	93	1	<0.01
Louisiana	64/662	23.5	18	0.04–0.51	45.35	96	2	<0.01
Virginia	1/90	1	1	0.00–0.06	-	-	-	-
Tennessee	55/860	6.4	6	0.05–0.08	-	-	-	-
Dog types								
Residential	145/1530	16.3	12	0.04–0.28	118.64	97	4	<0.01
Shelter	211/2150	7.9	7	0.04–0.12	71.29	92	6	<0.01
Working	104/644	39.9	32	0.07–0.74	122.63	99	1	<0.01
Stray/Feral	77/470	26.4	23	0.02–0.79	82.46	98	2	<0.01

### Canine *Trypanosoma cruzi* infection in different dog types

Prevalence differed notably across dog types, as working dogs exhibited the highest mean *T. cruzi* infection prevalence at 38.9%, followed by stray/feral dogs at 26.4% and residential dogs at 16.3%. Shelter dogs showed the lowest prevalence, with a mean estimate of 7.9% (Table 2). These patterns suggest variation in infection risk that may be associated with differing levels of outdoor exposure and housing conditions.

### Meta-analysis and subgroup analysis

**Figure 3:**
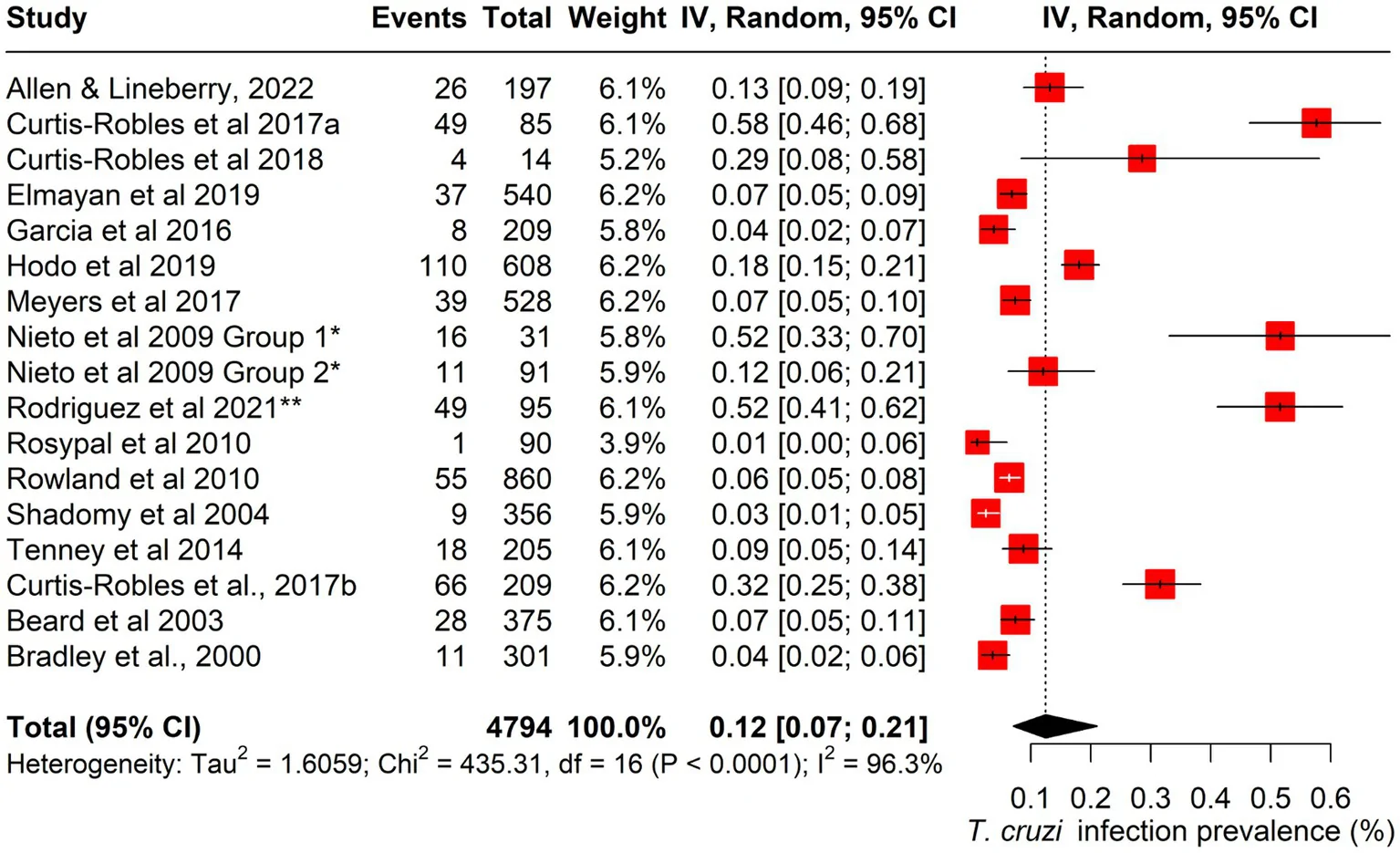
Forest plot showing the estimated pooled prevalence of Canine *T. cruzi* infection in the U.S.

**Figure 4:**
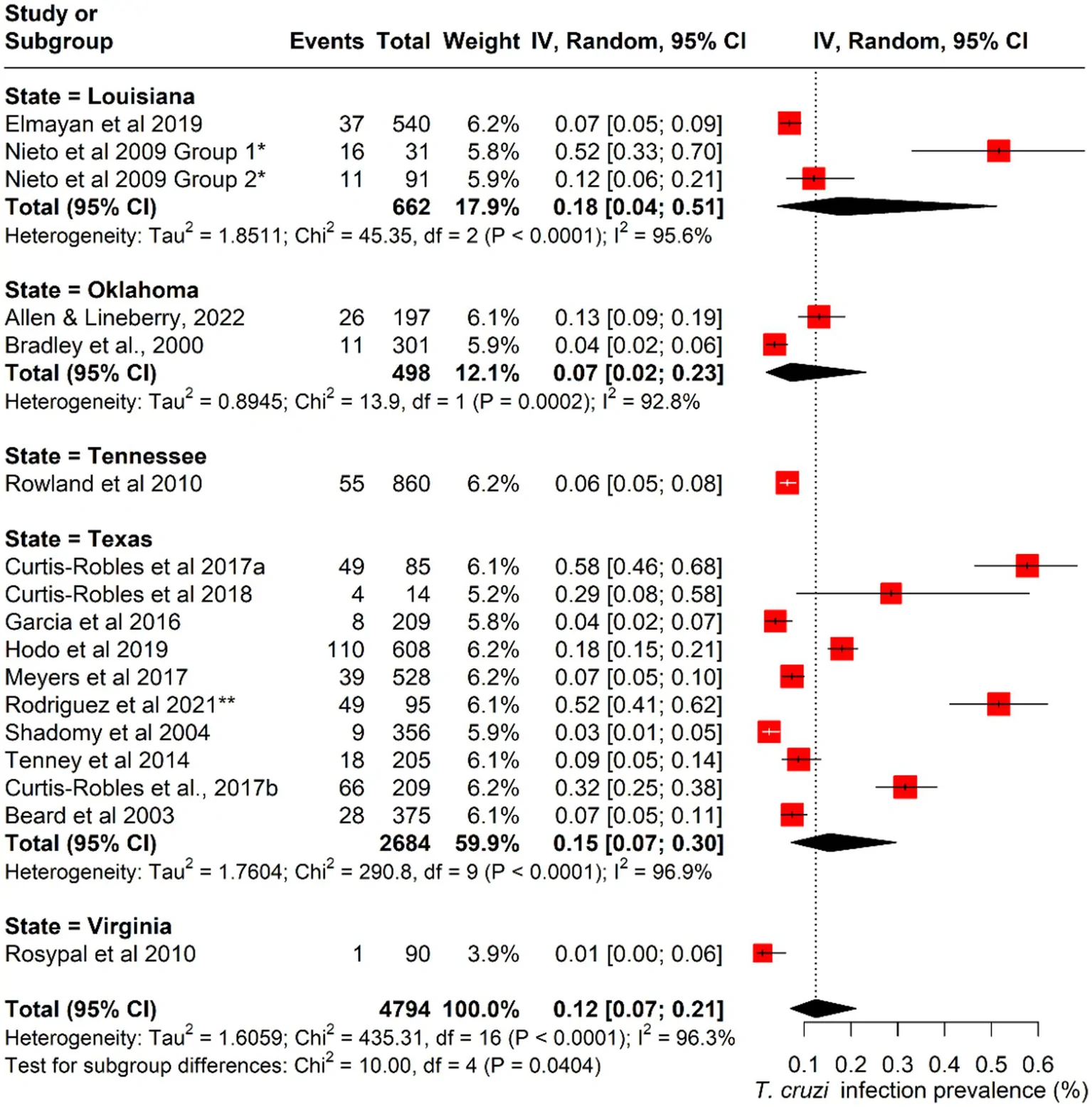
Forest plot of subgroup analysis of the prevalence of Canine *T. cruzi* infection across different states in the U.S.

**Figure 5:**
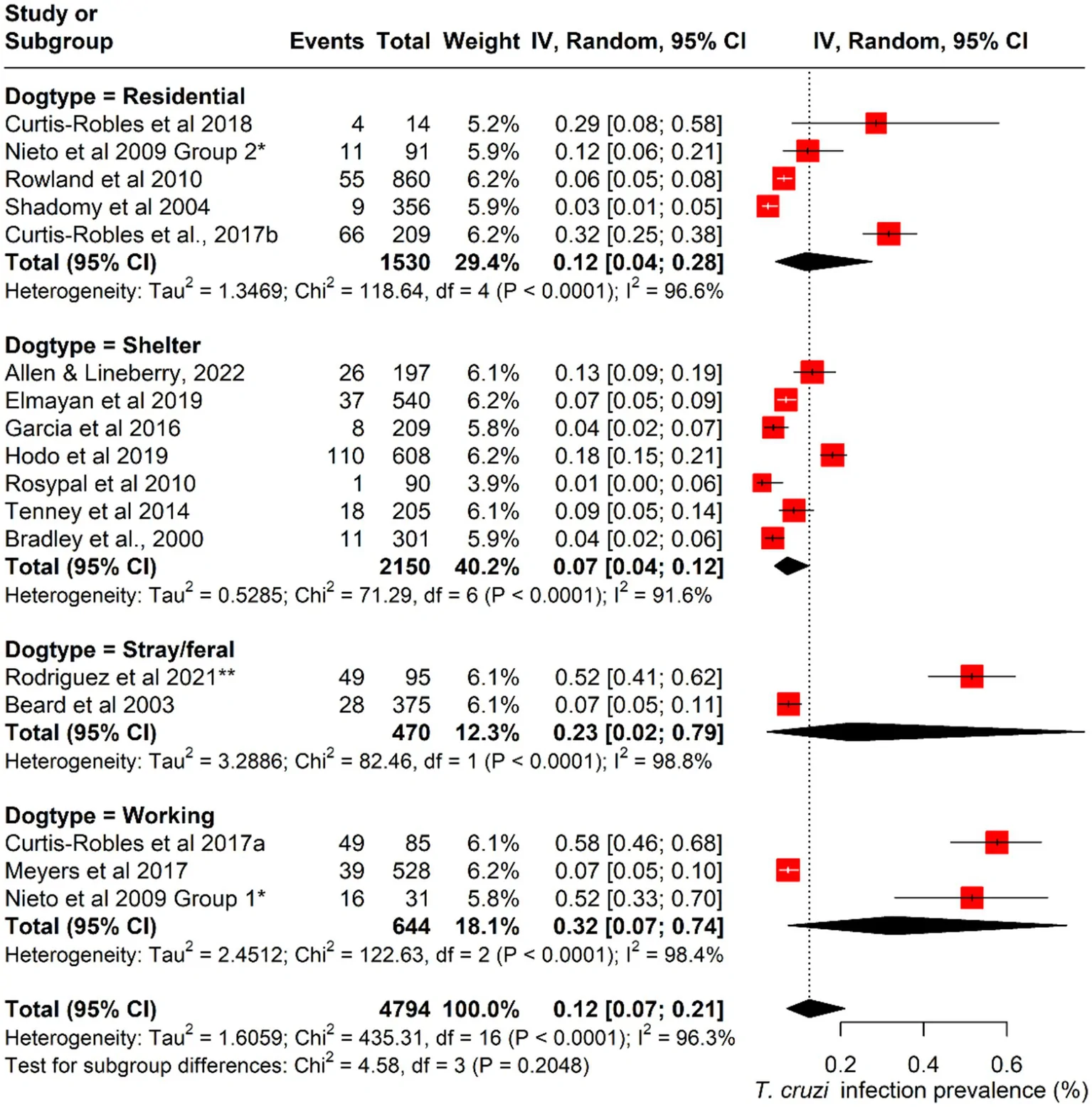
Forest plot of subgroup analysis of the prevalence of Canine *T. cruzi* infection across different dog types in the U.S.

The pooled prevalence of canine Chagas disease across all studies was estimated at 12.5% (95% CI: 0.07–0.21; *I^2^* = 96%; *Q* = 435; *p* < 0.001) (Figure 3). Subgroup analyses revealed significant variations by state and dog type. The pooled prevalence was also highest in Louisiana (15%; 95% CI: 0.04–0.51), followed by Texas (15%; 95% CI: 0.07–0.30%). Among different dog types, working dogs had the highest pooled prevalence (32%; 95% CI: 0.07–0.74), followed by stray/feral dogs (23%; 95% CI: 0.02–0.79) (Figures 4, 5; Table 2).

### Meta-regression analysis

The meta-regression indicated that study year was significantly associated with canine *T. cruzi* infection prevalence, with an estimated 11% increase in odds per year, suggesting either a real temporal rise or improved detection/reporting over time. Dog type and state did not significantly predict prevalence, likely reflecting limited study numbers and variability. Although moderators explained about 28% of between-study heterogeneity, residual heterogeneity remained very high (*I^2^* = 95%), indicating that additional unmeasured factors contribute to the variation across studies.

### Publication bias and sensitivity analysis

**Figure 6:**
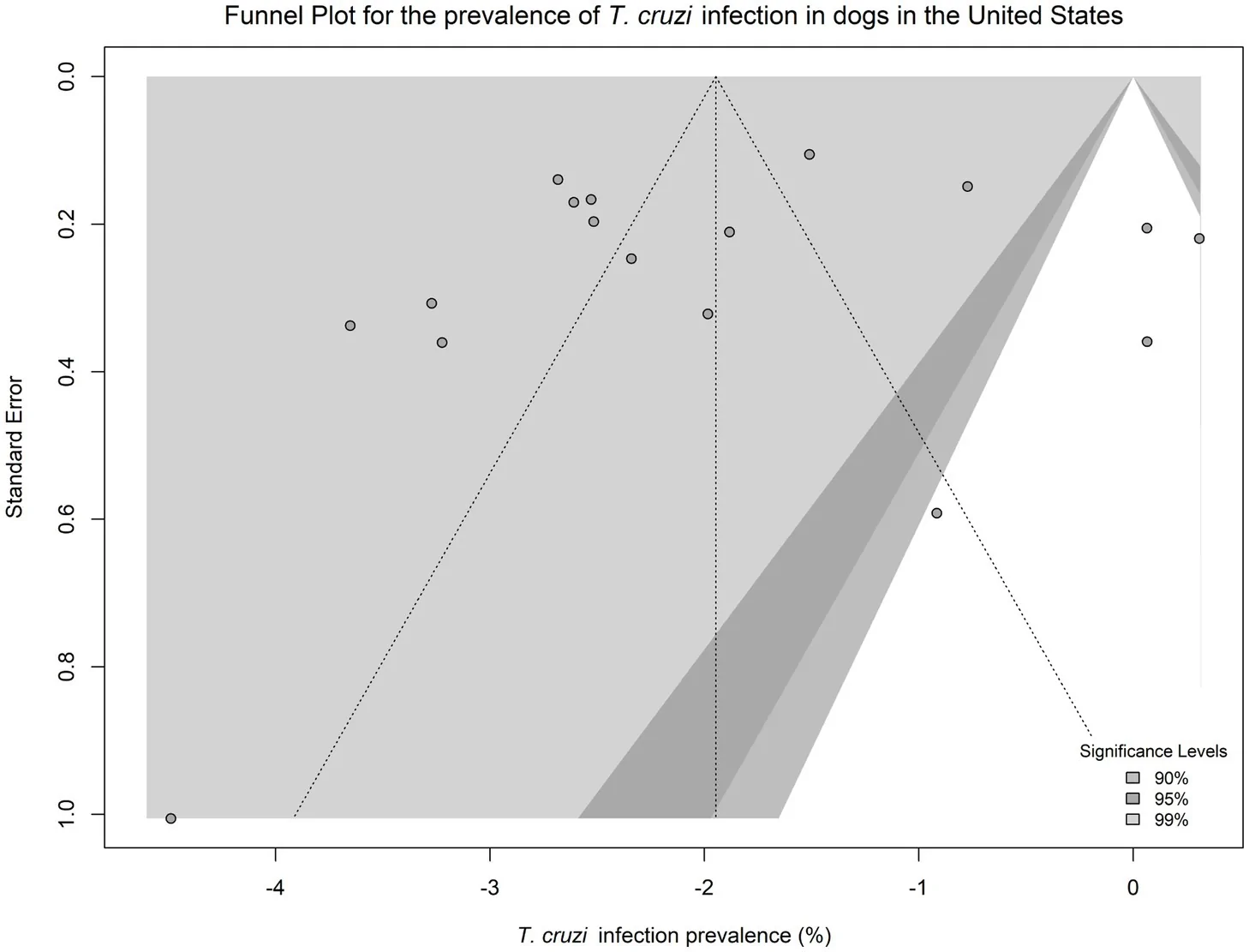
Funnel plot displaying the observed effect size of each study against the standard error of each study.

Egger’s test did not indicate evidence of publication bias (*p* = 0.470), and the funnel plot appeared symmetric (Figure 6). Sensitivity analysis revealed that no single study disproportionately influenced the pooled prevalence estimates, and heterogeneity remained high (*τ^2^* = 1.743, *I^2^* = 99.78%), and the pooled effect size was −2.087 (SE = 0.331, 95% CI: −2.737 to −1.438, *p* < 0.001).

## Discussion

This systematic review and meta-analysis provide new insights into the prevalence and epidemiological significance of *T. cruzi* infection in dogs in the U.S. The evidence indicates that most prevalence studies have been conducted in the southern U.S., consistent with the known distribution of triatomine vectors and the geographic focus of past research. This concentration of data, however, restricts our ability to evaluate prevalence patterns in other regions where environmental conditions could also sustain vectors and transmission cycles (26, 27). The limited scope of available studies underscores the need for expanded surveillance across a broader geographic range. Future investigations in northern Texas, the Midwest, and other understudied regions are particularly warranted given the potential for northward expansion of triatomine populations driven by climate and ecological changes (28). Wildlife reservoirs, including raccoons and opossums, already harbor *T. cruzi* across diverse habitats, suggesting that transmission cycles may exist outside historically recognized areas (29, 30).

In the southern U.S., housing infrastructure and triatomine ecology have likely reduced opportunities for human–vector contact, thereby lowering direct risk to people, however, this protective effect does not extend equally to dogs. In the U.S., transmission is primarily sylvatic rather than domestic, and canine risk is shaped more by outdoor activity and oral exposure than by indoor housing conditions. This is particularly relevant for working dogs housed in kennel, and other high-exposure dog types (31). States such as Tennessee and Virginia, which lie in the Upper South, already report *T. cruzi* infections in both dogs and wildlife, reinforcing the need to recognize these regions as part of the broader ecology of transmission. The lack of comprehensive, geographically diverse surveillance may therefore lead to underestimation of canine *T. cruzi* infection and, by extension, the risks of transmission to other hosts.

Broader geographic and seasonal surveillance of *T. cruzi* in dogs and triatomine vectors is necessary to identify emerging hotspots and refine risk assessments. Future research should also focus on characterizing the spatial distribution of *T. cruzi* based on habitat type to better understand its transmission dynamics in sylvatic, peri-domestic, and urban settings. Expanding surveillance efforts will help identify high-risk areas, improve disease control strategies, and enhance our understanding of *T. cruzi* transmission beyond traditionally endemic regions. The overall prevalence of *T. cruzi* infection in dogs across different states reinforces their potential role as important reservoir hosts in the U.S. (32).

However, the substantial heterogeneity observed in this review is likely attributable to differences in study design, sampling strategies, and particularly the serological assays employed; hence, pooled infection prevalence estimates should be interpreted with caution. Studies using only one serological assay may either overestimate prevalence due to cross-reactivity or underestimate it if assay sensitivity is limited. The lack of a universally accepted serological gold standard for canine *T. cruzi* infection remains a major limitation in prevalence research. Standardized diagnostic protocols, ideally incorporating dual-assay confirmation, would substantially improve the reliability and comparability of future studies. Until such harmonization is achieved, heterogeneity in prevalence estimates will persist, underscoring the need for careful interpretation when synthesizing findings across diverse datasets.

Observed differences in infection prevalence across states may partly reflect the distribution and ecology of specific triatomine species rather than true differences in canine infection risk alone. For example, *Triatoma gerstaeckeri*, reported primarily from Texas, has been documented with relatively high *T. cruzi* infection proportions and is frequently collected in peridomestic settings and dog kennels (31). However, because vector surveillance and reporting were not consistent across the included studies, species-level inferences should be interpreted cautiously. Within the subset of southern states represented in our dataset, higher infection prevalence estimates were frequently reported in Louisiana and Texas, whereas lower estimates were reported from Oklahoma, Tennessee, and Virginia. These patterns likely reflect a combination of factors, including uneven sampling effort, variable study designs, differences in dog types sampled, assay heterogeneity, and environmental conditions that influence vector distributions. Importantly, we did not conduct statistical comparisons across states with markedly different sample sizes or across dog types represented by few studies, as such contrasts could be misleading. Instead, we present pooled estimates with confidence intervals to convey uncertainty and acknowledge variability.

Given these limitations and the lack of standardized, study-level vector search methods, vector information was treated descriptively rather than analyzed as a moderator of infection prevalence. Triatomine detections reported in individual studies should not be interpreted as definitive evidence of presence or absence at the state level. In addition, wildlife reservoirs (e.g., raccoons, opossums, and other synanthropic mammals) likely contribute to local transmission dynamics in sylvatic and peridomestic settings, potentially influencing canine exposure, but their effects could not be quantified in this review (17). Overall, the geographic patterns presented here should be regarded as provisional and dependent on the current, inherently heterogeneous evidence base.

Variation by dog type likely reflects differences in exposure opportunity rather than inherent susceptibility. Working dogs (e.g., patrol, detection, search-and-rescue) often spend prolonged time outdoors, are frequently housed in peridomestic kennel settings, and may train at dusk or night—all conditions that plausibly increase contact with triatomines and contaminated environments (5, 9, 12, 13). Stray/feral dogs, which are free-roaming with minimal veterinary oversight, spend most of their time outdoors at the sylvatic–peridomestic interface and may encounter vectors in sleeping sites, dens, or while scavenging (32, 33). Residential dogs showed intermediate estimates, consistent with heterogeneous husbandry (indoor/outdoor access, yard sleeping, porch lighting, etc.). Shelter dogs had the lowest infection prevalence estimates; however, this finding should be interpreted with caution. Shelter populations are heterogeneous, often including former strays, owner surrenders, and dogs with unknown exposure histories, and housing conditions vary widely across facilities (e.g., indoor vs. outdoor kennels). Moreover, some categories in this review were represented by few studies, and dog-type effects may be confounded by geography, as several working and stray/feral cohorts were sampled in higher-prevalence states. Monitoring and control efforts may therefore need to prioritize high-risk populations, particularly feral, working, and military dogs, given their critical functional roles and heightened susceptibility to infection.

Information on triatomines was inconsistently collected and reported across studies, limiting the ability to assess their influence on canine infection prevalence. Where reported, recognized vectors such as *Triatoma gerstaeckeri*, *T. sanguisuga*, *T. rubida*, and *T. protracta* were documented in the same states as sampled dogs, and prior work has shown species-level differences in ecology and infection proportions that could shape local exposure risk (23, 31, 34). However, vector surveillance methods varied considerably: some studies included active searches (e.g., Rodriguez et al. (25), others relied on incidental findings, and several did not assess vectors at all). As a result, triatomines were documented in Texas, Louisiana, and Oklahoma, while none were reported in Tennessee or Virginia. This absence should not be interpreted as evidence of true absence, as all states included in this review fall within the known geographic range of triatomine vectors (23, 25, 31, 34, 35). Because of this heterogeneity in methods and reporting, vector presence was not analyzed as a moderator in the meta-analysis and is instead presented descriptively to provide context for interpreting prevalence findings. Our findings on vector occurrence should therefore be interpreted with caution, and future work should incorporate standardized, concurrent entomological surveillance alongside canine sampling to better evaluate species-specific contributions to transmission risk.

Meta-regression identified dog type as the only significant moderator of prevalence, but it explained just 8.1% of the overall heterogeneity. This indicates that the most variability between studies is likely driven by unmeasured factors not captured in the selected moderators. State was not a statistically significant predictor, likely due to the limited number of studies per state and the uneven sampling effort across regions. These findings emphasize the multifactorial nature of *T. cruzi* transmission, which reflects a complex interplay of host management, vector ecology, and environmental conditions rather than a single dominant driver.

The interpretation of pooled prevalence estimates must therefore be approached with caution. Texas contributed 10 of the 16 studies and more than half of the total sampled dogs, while states such as Virginia were represented by only a single small-scale study. Similarly, classification inconsistencies required the merging of stray and feral dogs into a single group, which may have diluted distinctions between these populations. Although the random-effects model accommodates such heterogeneity, it cannot fully resolve the limitations posed by uneven geographic coverage and imbalanced sample sizes. For this reason, pooled estimates in this review should be regarded as indicative patterns rather than definitive measures of national prevalence.

Egger’s test did not suggest publication bias, and sensitivity analysis showed that no single study disproportionately influenced pooled estimates. Nonetheless, the high heterogeneity observed across studies underscores the importance of context-specific interpretation. Future research should integrate ecological, spatial, and genetic data with standardized diagnostic approaches to refine risk assessments. Broader surveillance outside historically recognized regions, coupled with the use of combined serological and molecular methods, will help generate more reliable prevalence estimates and support targeted interventions tailored to local epidemiological conditions.

### Study limitations

This study has several limitations that should be considered when interpreting the findings. Firstly, our analysis relied on published studies, and negative or non-significant results may be underrepresented in the literature, while Egger’s test did not detect strong evidence of bias, the potential remains. Secondly, most of the included studies were conducted in Texas, with very few studies from other states, more so, some states had only a single small dataset (e.g., Virginia, Tennessee), while large areas of the U.S. where triatomine vectors are known to occur had no eligible studies at all. This imbalance restricts the generalizability of our pooled estimates and prevents robust comparisons across regions. Meanwhile, a study by Meyers et al. (10) retained in this analysis could not be stratified by state due to security-sensitive sampling information of some government working dogs. These were retained but reported as single entries, which limits geographic resolution. This constraint should be considered when interpreting state-level patterns.

Moreover, certain dog types (e.g., stray/feral) were represented by only one or two studies, limiting the reliability of subgroup estimates. Furthermore, inconsistencies in classification, such as the overlap between feral and shelter populations, likely introduced variability into pooled estimates. For example, shelter populations may include previously feral or stray dogs, complicating clear distinctions between categories. Several large-scale or recent studies were excluded because they did not meet inclusion criteria. For instance, Meyers et al. (12) reported prevalence from more than 1,600 dogs nationwide but did not provide state-level stratification, and Pace & Oppong (36) obtained samples from four veterinary facilities, two private practices in Denton and Dallas Counties and two additional clinics in Dallas County operated by the same corporate group, representing a convenience sample rather than a population-based survey. While outside the scope of our systematic framework, these studies nonetheless contribute important epidemiological insights and highlight gaps in geographically stratified surveillance.

Meta-regression identified dog type as a significant moderator, but this explained only a small portion of heterogeneity. Other relevant factors, such as breed, age, management practices, and environmental exposures, were not consistently reported across studies and therefore could not be analyzed. Our analysis did not account for local environmental variables such as vegetation type, wildlife host abundance, or land use, all of which are known to shape triatomine distributions and infection risk. The absence of such data limits our ability to interpret geographic differences in prevalence.

Finally, because all included studies were conducted within the known triatomine range in the southern U.S., our findings cannot confirm whether prevalence differs between southern and northern regions. This concentration underscores a substantial surveillance gap in areas outside historically recognized endemic zones. All these limitations highlight the need for more geographically diverse, systematically designed studies that use standardized dog type definitions, incorporate ecological variables, and combine serological and molecular diagnostics to improve the accuracy and comparability of prevalence estimates.

## Conclusions and recommendations

This systematic review and meta-analysis demonstrate that canine *T. cruzi* infection is present in the U.S., with most studies conducted in southern states such as Texas and Louisiana. However, these findings must be interpreted cautiously, as data are geographically limited, unevenly distributed, and methodologically heterogeneous. Differences in prevalence between dog types likely reflect variation in exposure opportunities, working and stray/feral dogs showing higher estimates, while shelter dogs generally showed lower prevalence, but subgroup interpretations remain tentative given limited and inconsistent data.

The observed heterogeneity underscores the need for standardized diagnostic protocols, clearer dog type classifications, and concurrent vector surveillance to improve comparability and reliability of prevalence estimates. Expanding surveillance into underrepresented regions, particularly the Midwest and northern states where vectors are known to occur, will be essential to better define the geographic distribution of infection. Preventive strategies should prioritize high-risk dog types through improved kennel management, vector control, and targeted screening, while community-based approaches will be needed for stray and feral dogs.

Future research should adopt interdisciplinary approaches that integrate serological and molecular diagnostics, ecological and spatial data, and incidence studies in defined dog types. Such efforts will refine risk assessments, strengthen surveillance, and inform context-specific interventions to mitigate the veterinary and public health impact of canine Chagas disease in the U.S.

## Data Availability

The data analyzed in this systematic review were derived from publicly available scientific literature databases containing article abstracts and full texts. All data used are in the public domain, and no proprietary or unpublished data were employed. Further details are available from the corresponding author upon reasonable request.
