# Correction: Prevalence of *Trypanosoma cruzi* infection in dogs in the United States: a systematic review and meta-analysis of observational studies (2000–2024)

**DOI:** 10.3389/fvets.2026.1979593

**Published:** 2026-09-16

**Authors:** Victor Agbajelola, Walter Roachell, Joshua Bast, Paul Lenhart, Mauricio Solis, Ram K. Raghavan

**Affiliations:** 1 Department of Pathobiology and Integrative Biomedical Sciences, University of Missouri, Columbia, MO, United States; 2 Public Health Command West, Joint Base San Antonio (JBSA), San Antonio, TX, United States; 3 Entomological Sciences Division, Defense Centers for Public Health - Aberdeen, Aberdeen, MD, United States; 4 Public Health Command Central, Joint Base San Antonio (JBSA), San Antonio, TX, United States; 5 Department of Public Health, College of Health Sciences, University of Missouri, Columbia, MO, United States; 6 MU Institute of Data Science and Informatics, University of Missouri, Columbia, MO, United States

**Keywords:** American trypanosomiasis, canine Chagas infection, kissing bugs, *Trypanosoma cruzi*, United States, working dogs

An incorrect number was provided for U.S. Army Contracting Command. “(Award # W911S expressed R2210004)” should be “(Award # W911SR2210004)”. The Funding statement has been corrected.

An Author's Disclaimer was erroneously omitted from the article. The Author's Disclaimer should read as, “Any opinions, findings, and conclusions or recommendations expressed in these materials are those of the author(s) and do not necessarily reflect the position or policy of the Government, and no official endorsement should be inferred”.

The original version of this article has been updated.

